# Bridging the Gap∗

**DOI:** 10.1016/j.jaccas.2023.101979

**Published:** 2023-08-23

**Authors:** Brian Whisenant, Alec Vahanian

**Affiliations:** a Intermountain Medical Center, Murray, Utah, USA; b University Paris-Descartes, Paris, France

**Keywords:** mitraclip, mitral stenosis, mitral valvuloplasty, rheumatic mitral stenosis, TEER

**Figure:**
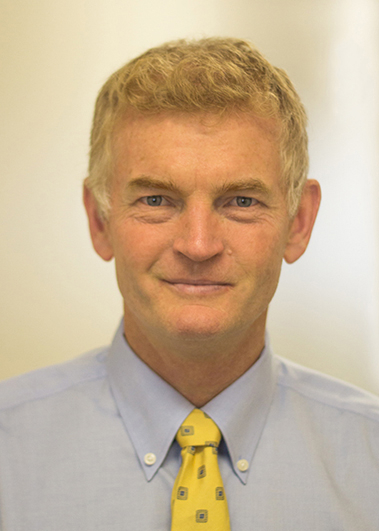

When first introduced some 40 years ago, percutaneous balloon mitral commissurotomy (PBMC) in patients with rheumatic mitral stenosis (MS) and favorable anatomy was recognized as an alternative to surgical commissurotomy providing comparable hemodynamic and durable results.^1^, ^2^, ^3^ As PBMC found its role compared with well-established surgery, an extensive literature identified anatomic predictors of safety and efficacy with PBMC.^4^^,^^5^ PBMC enjoys a Class 1a indication for patients with severe rheumatic MS and favorable valve morphology when performed at a comprehensive valve center, whereas surgery has a 1B indication for severe rheumatic MS in patients who are not candidates for PBMC, have failed a previous PBMC, require other cardiac procedures, or do not have access to PBMC.^6^

Recent publications from Europe and North America demonstrate that compared with prior decades, patients currently treated with PBMC are older, have less favorable mitral valve characteristics for PBMC, and have more complications.^7^^,^^8^ PBMC with suboptimal anatomy may be complicated by leaflet tears, papillary muscle rupture, and chordal rupture and require surgical mitral valve reconstruction or replacement.^9^ Perhaps because results are less gratifying, there are few publications regarding the outcomes of PBMC in patients with unfavorable anatomy.^10^ The guidelines support PBMC for highly symptomatic patients with severe rheumatic MS and suboptimal anatomy who are not candidates for surgery or are at high risk for surgery with a Class IIb indication.^6^

Beyond rheumatic MS, calcific MS is a different entity that occurs when mitral annular calcification extends into the mitral leaflets, narrowing the annulus and limiting leaflet mobility.^11^ Calcific MS is not only observed in elderly patients with numerous comorbidities but is inherently challenging for the surgeon with risks of atrioventricular groove disruption, paravalvular mitral regurgitation, and excess mortality. Many patients with calcific MS are evaluated for transcatheter mitral valve replacement, but most are deemed anatomically unsuitable with current technologies.^12^ Cardiologists are thus left with patients with highly symptomatic calcific MS and may turn to high-risk PBMC as a palliative option of last resort without supportive literature.

In this issue of *JACC: Case Reports*, Dowling et al^13^ describe a 66-year-old woman with severe rheumatic MS, class IV heart failure, more than 10 heart failure admissions, extreme operative risk secondary to numerous comorbidities, and suboptimal anatomy for PBMC. Mitral valvuloplasty resulted in a tear of the medial A2 leaflet resulting in severe mitral regurgitation (MR) and unstable hemodynamics. When faced with torrential MR and hemodynamic extremis, a MitraClip NT (Abbott Laboratories) was deployed across the torn leaflet, resulting in mild to moderate residual MR and a mean gradient of 9 mm Hg. The patient was able to undergo surgical mitral valve replacement 5 days later and was ultimately discharged home on postoperative day 7.

This case report is to our knowledge the first reported of the use of transcatheter edge-to-edge repair (TEER) for the treatment of a severe MR after PBMC. TEER predictably reduces mitral valve area and is therefore contraindicated with rheumatic mitral valve disease and MS.^14^ In the reported case, balloon-associated mitral leaflet fracture occurred when commissural calcification and rigid fibrosis prevented commissural opening, and balloon inflation tore the less fortified leaflet. An example of a similar complication is shown in Figure 1. Without commissural opening, MS predictably ensued. However, the creative TEER placement across the anterior leaflet tear rather than bridging the anterior and posterior leaflets preserved the small residual valve area.

**Figure 1:**
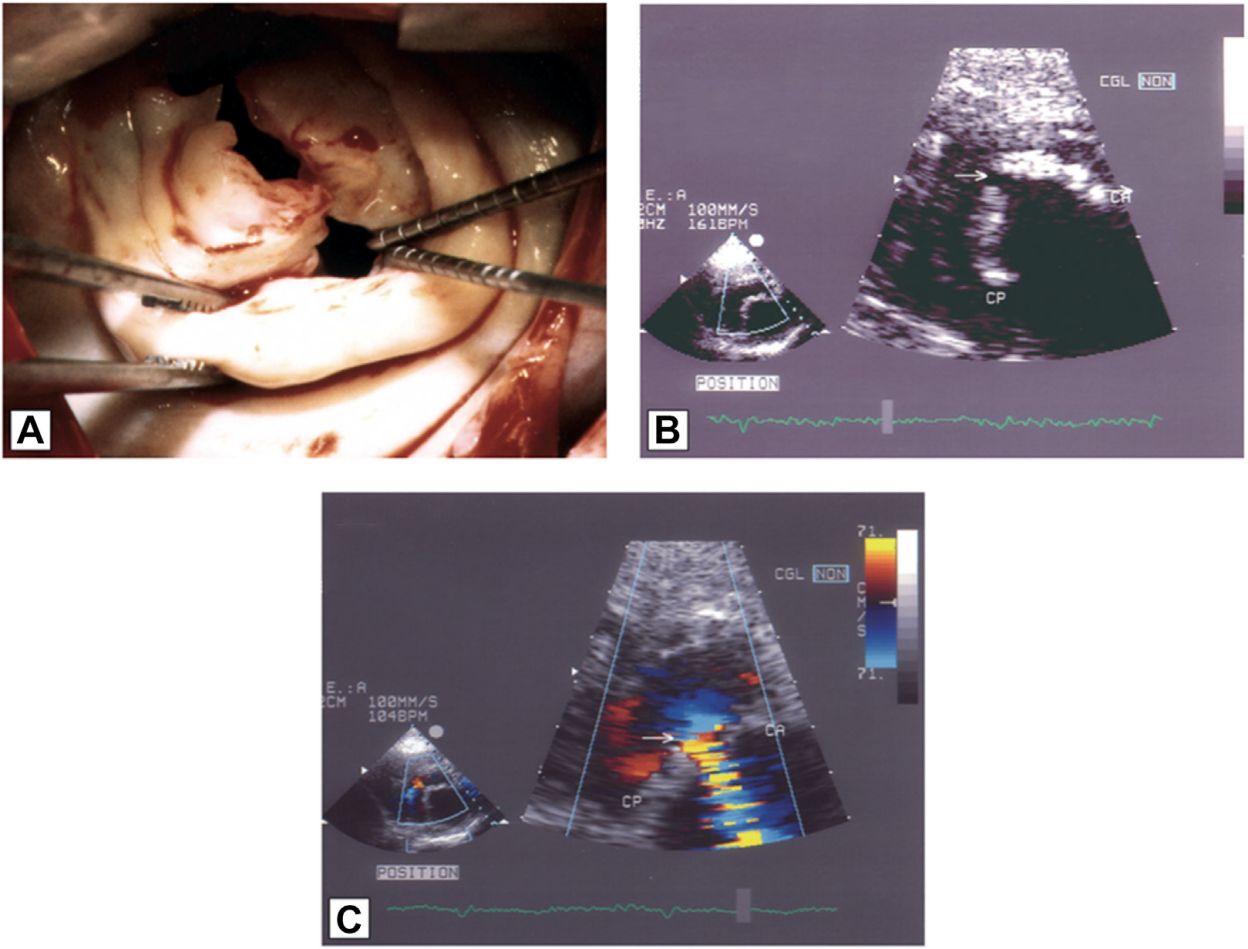
Example of Anterior Leaflet Tear Following Percutaneous Balloon Mitral Commissurotomy in a Patient With Calcified Mitral Stenosis **(A)** Intraoperative view showing a tear in the mid part of the anterior mitral leaflet. **(B)** Transthoracic echocardiography showing the tear of the anterior mitral leaflet. **(C)** Color Doppler confirming severe mitral regurgitation. Adapted from Vahanian A. Percutaneous balloon mitral commissurotomy. In The PCR-EAPCI Textbook, Europa Publishing.

In addition to highlighting a novel and lifesaving single-leaflet use of a device designed for edge-to-edge repair, this case demonstrates a highly functioning collaboration in a comprehensive valve center consistent with recent guidelines. Imaging experts defined the etiology and severity of the valve disease. The case was discussed in a heart team meeting in which PBMC was recommended. When faced with a potentially catastrophic complication, an emergency heart team meeting was convened. A highly technical ad hoc salvage TEER was performed. After stabilization of the clinical status, faced with no good options, surgeons accepted the challenge of emergency mitral valve repair in a high-risk patient, and despite numerous comorbidities, the patient not only survived surgery but was managed through her hospitalization until discharged to home.

Structural cardiologists, armed with new tools and creative experience are today faced with and are tackling high-risk procedures in deserving patients who very recently would have been palliated with oxygen and diuretics. Among such patients, MS in the elderly is commonly encountered, often disabling, and difficult to manage. The accompanying report not only details an important rescue single-leaflet use of a device designed for edge-to-edge repair but is an example framework of heart team collaboration to meet this unique patient’s medical need.

## Funding Support and Author Disclosures

Dr Whisenant is a consultant for Abbott Laboratories and Edwards Lifesciences. Dr Vahanian is a consultant for VenusMedTech; has received speaker honoraria from Edwards Lifesciences; and is Emeritus professor Université Paris Cité, Paris France.
